# Evasin and TSLPI Tick Salivary Antigen Subunit Vaccine Nanoparticles Induce Humoral and Cellular Immunity

**DOI:** 10.1021/acsnanoscienceau.5c00034

**Published:** 2025-08-12

**Authors:** Jaeyoung Park, Thomas Pho, Stepan S. Denisov, Ingrid Dijkgraaf, Julie A. Champion

**Affiliations:** † School of Chemical and Biomolecular Engineering, 1372 Georgia Institute of Technology, 950 Atlantic Dr. NW, Atlanta, Georgia 30332, United States; ‡ Bioengineering Program, 1372 Georgia Institute of Technology, Atlanta, Georgia 30332, United States; § Department of Biochemistry, Cardiovascular Research Institute Maastricht (CARIM), 5211 Maastricht University, Maastricht 6229 ER, Netherlands

**Keywords:** tick vaccine, tick salivary proteins, Evasin-3, TSLPI, subunit vaccine, nanoparticles, desolvation

## Abstract

Tick-borne diseases have increased significantly due to several factors, including climate change. Ticks can carry diverse pathogens, and transmission is facilitated by immunosuppressive tick salivary proteins. Vaccination targeting tick salivary proteins has been proposed as a strategy to enhance broad acquired immunity against tick-borne pathogens. Given the immunosuppressive nature of these proteins, we leveraged the ability of nanoparticles to enhance antigen immunogenicity. We synthesized nanoparticles directly from tick salivary proteins, evasin-3 and tick salivary lectin pathway inhibitor (TSLPI), by desolvation with ethanol and cross-linking. Nanoparticles formulated with the CpG oligonucleotide adjuvant significantly enhanced both humoral and cellular immune responses against both evasin-3 and TSLPI in mice compared to soluble CpG adjuvanted antigens. These results demonstrate the importance of antigen delivery and presentation, particularly for poorly immunogenic antigens, and the potential for protein nanoparticles to be developed as vaccines against diverse tick-borne pathogens.

## Graphical abstract

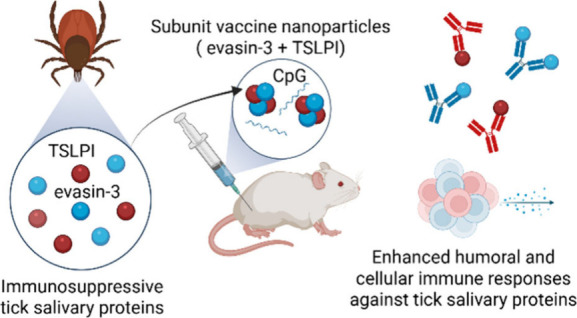

## Introduction

In recent years, increases in tick-borne diseases have been reported, especially in North America, partly due to climate change greatly expanding the range of tick prevalence to longer seasons and new areas.^1^ One of the most reported tick-borne diseases is Lyme disease, which accounts for >36,000 cases reported to the Center for Disease Control and Prevention in the United States each year, though recent statistics based on insurance records indicate that around 500,000 individuals are treated for this disease annually.^2–5^ Other emerging tick-borne diseases include Anaplasmosis, Erhlichioisis, and Babesiosis, which have rapidly increased in incidence. While prophylactic antibiotics remain effective against Lyme disease following a known tick bite, antibiotics may not be sufficient for the prevention of Babesiosis, a fatal tick-borne disease.^6^ Vaccines are a safe and effective approach to protect against pathogens; however, there are currently no commercially available vaccines against multiple emerging tick-borne diseases. VLA15 by Valneva and Pfizer is a Lyme disease vaccine candidate currently in a phase 3 clinical trial. VLA15 is a subunit protein vaccine based on the outer surface protein A (OspA) of *Borrelia burgdorferi*, allowing antibodies to block the bacteria’s ability to leave the tick.^7^

Unfortunately, multiple pathogens with significant diversity exist in ticks,^8–10^ limiting the effectiveness of pathogen-specific vaccine formulations. Furthermore, even when a vaccine is demonstrated to be effective against a tick-borne disease, the formulation and choice of vaccine targets for broad application are critical considerations. These factors can influence public perception and demand, ultimately affecting commercial success. For example, LYMErix, an anti-Lyme disease vaccine, was shown to be effective but was withdrawn from the market due to poor sales and a lack of demand.^11,12^ This was largely because the vaccine was recommended only for individuals living in areas with a high risk of Lyme disease, restricting its broader adoption. This spurs tick vaccine development toward formulating broadly responsive vaccines with tick salivary proteins.^13–15^ Tick saliva is a rich source of immunosuppressive compounds that facilitate transmission of various tick pathogens by interfering with the host immune system.^16–18^ Targeting these proteins instead of specific pathogen antigens could induce the host immune system to effectively prevent infection by multiple different tick-borne pathogens. While identification of tick antigens is significant for vaccine development, vaccine formulation and delivery are equally crucial since, to date, immunization with a single tick salivary antigen cannot attain tick immunity commensurate to naturally acquired tick immunity.^13^ Sajid et al. reported an mRNA-lipid nanoparticle (NP) vaccine encoding 19 tick salivary proteins that effectively hampered tick feeding and inhibited *B. burgdorferi* transmission in guinea pigs.^15^ This work establishes that immunization with several immunogenic tick salivary proteins can result in strong prophylactic activities against the tick-borne pathogen, although the minimum number is not known. Additionally, the vaccine platform itself can also significantly affect the onset of tick immune responses.^13,19^ Therefore, it is important to consider antigen delivery systems for tick vaccine development. NPs alter cell uptake and tissue retention properties of antigens, serve as adjuvants, and present multivalent display of antigens on the surface, which may all contribute to increased immunogenicity.^20,21^ In previous work, we demonstrated that protein NPs made from desolvated antigen proteins enhanced adaptive immune responses against pathogens, including influenza and tick-borne *Orientia tsutsugamushi*.^22–25^ This approach was applied to the development of tick vaccines using two tick salivary proteins.

In this study, we used immunosuppressive evasin-3 and tick salivary lectin pathway inhibitor (TSLPI) proteins, with sequences from *Rhipicephalus sanguineus* and *Ixodes scapularis*, respectively, to form antitick vaccine NPs. Evasin-3 impedes tick immunity by impairing CXCL8-induced chemotaxis of neutrophils,^16^ while TSLPI inhibits lectin pathway complement activation.^18^ Based on the premise that adjuvants improve immunogenicity of weak antigens,^26–28^ we incorporated CpG oligonucleotide (ODN) 1826 in the NP formulation and demonstrated that the adjuvanted tick antigen NP vaccine incorporating evasin-3 and TSLPI antigens elicited stronger humoral and cellular responses compared to a soluble adjuvanted mixture of evasin-3 and TSLPI. We speculate that this simple vaccine formulation would be a promising approach to combat the rising number tick borne diseases.

## Results and Discussion

Evasin-3 and mTSLPI proteins were produced in *E. coli* and purified. As TSLPI retained the N-terminal methionine required for *E. coli* expression, it is heretofore referred to as mTSLPI. Purity and molecular weights of evasin-3 and mTSLPI were analyzed by high-performance liquid chromatography (HPLC) and electrospray ionization spray-mass spectrometry ESI-MS (Figures 1A,B, and S1). HPLC and ESI-MS reveal a single peak for each protein, indicating high purity of evasin-3 and mTSLPI. Deconvolution of ESI-MS confirms the molecular weights of 7005 and 8142 Da for evasin-3 and mTSLPI, respectively (Figure 1B), aligning with their calculated values.

**Figure 1:**
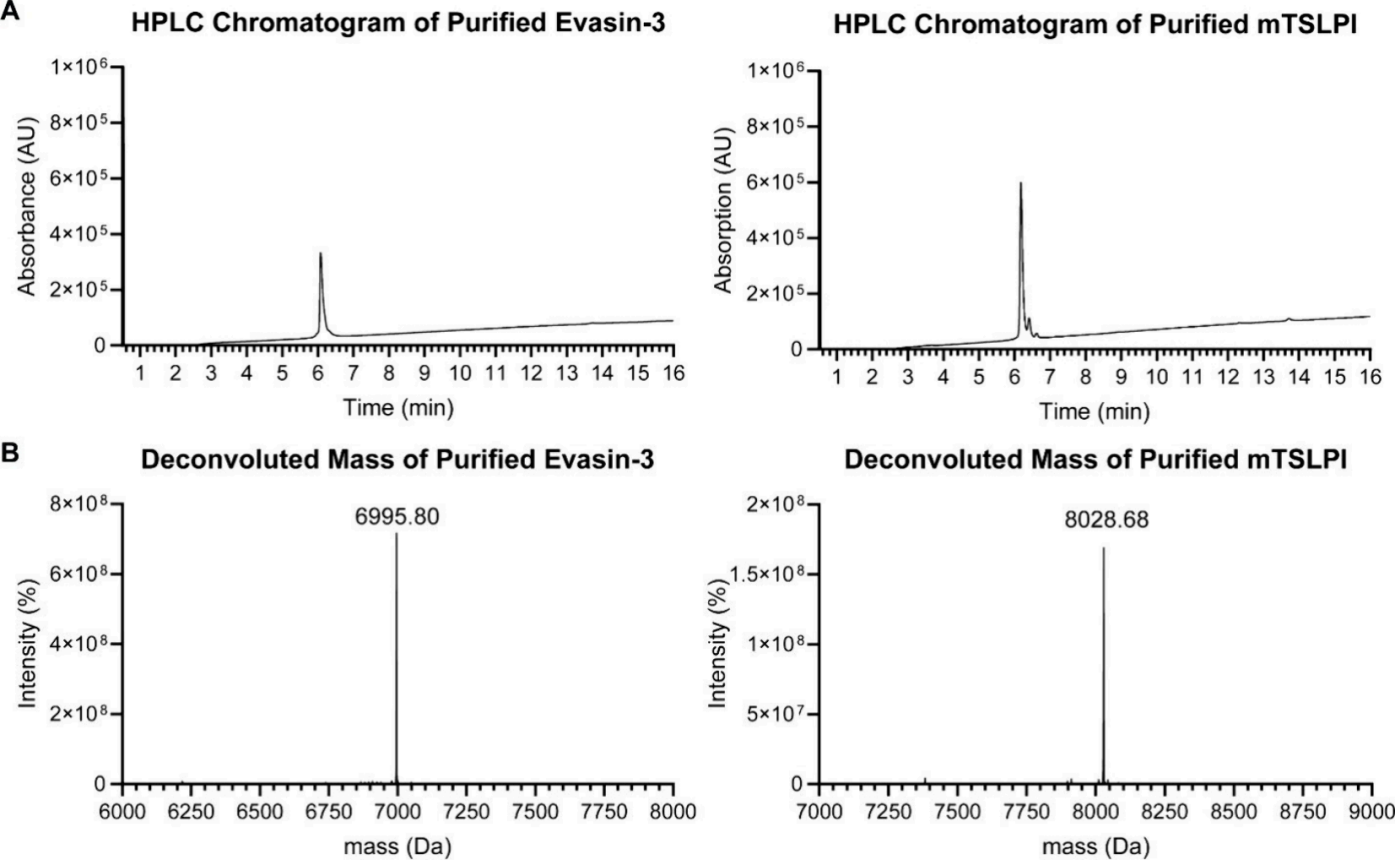
Analysis of produced and purified evasin-3 and mTSLPI proteins. (A) HPLC analysis of protein purity.

NPs were synthesized from tick salivary antigens by desolvation, as illustrated in Figure 2A. Ethanol desolvant was added dropwise to induce hydrophobic clustering of evasin-3 and mTSLPI antigens in phosphate-buffered saline (PBS). NPs were stabilized using 3,3′-dithiobis-sulfosuccinimidyl propionate (DTSSP), a reducible bifunctional NHS-ester cross-linker. The hydrodynamic diameters of single antigen evasin NP and mTSLPI NP were ∼330 and ∼500 nm, respectively, as measured by DLS (Dynamic Light Scattering) along with polydispersity index (Figure 2A,B). When both evasin and mTSLPI were formulated together into NPs at a 1:1 mass ratio, the size was ∼240 nm, though with higher polydispersity than single antigen NPs. The surface charge of each NP formulation was determined by measuring zeta potential. Evasin NP and mTSLPI NP had a charge of −40.8 mV and −12.3 mV, respectively. The surface charge of evasin/mTSLPI NP was −24.7 mV, which falls between the surface charges observed for a single antigen NP (Figure 2C). This suggests that both evasin and mTSLPI incorporated in evasin/mTSLPI NP contributed comparatively to the shift in the NP surface characteristics. Transmission electron microscopy (TEM) images were also taken for each NP (Figure 2D). Evasin NP, mTSLPI NP, and evasin/mTSLPI NP displayed generally spherical morphologies, with some irregularities. Since evasin/mTSLPI NP yielded the smallest NP size and contained both evasin and mTSLPI, they were used as the vaccine formulation for *in vivo* evaluation.

**Figure 2:**
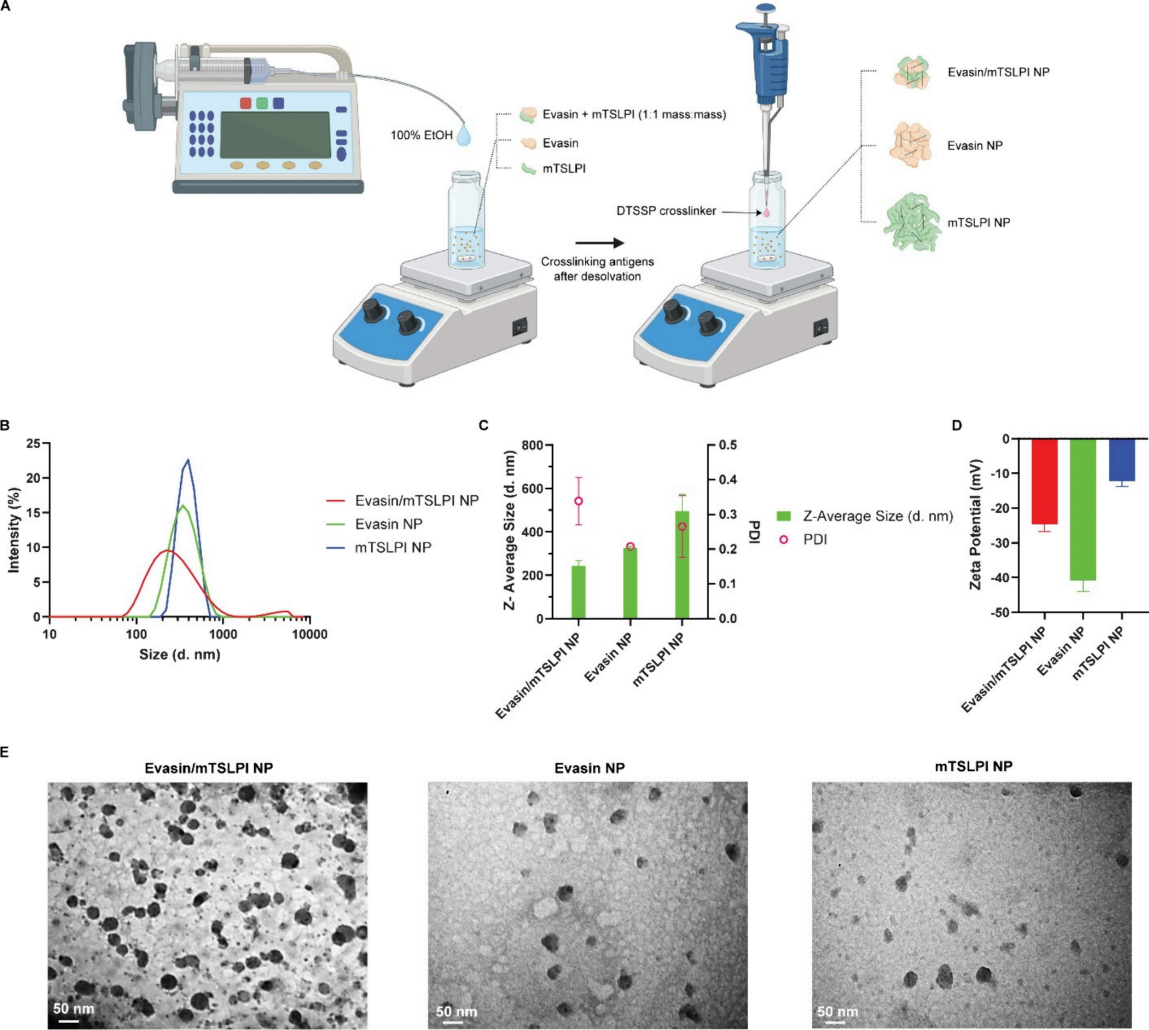
Synthesis and characterization of tick vaccine nanoparticles. (A) Schematic illustration of tick vaccine nanoparticle fabrication. Evasin/mTSLPI NP, evasin NP, and mTSLPI NP were synthesized by desolvating 1:1 mass ratio mixture of evasin and mTSLPI, evasin only, or mTSLPI only, respectively, followed by stabilization with DTSSP cross-linker. Created with BioRender.com. (B) Size distribution of evasin/mTSLPI NP, evasin NP, and mTSLPI NP. (C, D) Measurement of (C) size (diameter), polydispersity, and (D) zeta potential of NPs. (E) Representative TEM images of evasin/mTSLPI NP, evasin NP, and mTSLPI NP.

To assess the immune responses elicited by NPs composed of tick salivary proteins, we intramuscularly vaccinated mice with evasin/mTSLPI NP with and without CpG adjuvants, evasin/mTSLPI soluble mixture with and without CpG adjuvants, and PBS as a control. A boost injection was given on day 28 (Figure 3A). Body weight loss was not observed in mice within 5 days postvaccination (Figure S2). We first determined the magnitude of the improved humoral immune response induced by evasin/mTSLPI NP and evasin/mTSLPI NP + CpG. Notably, in contrast to evasin/mTSLPI and evasin/mTSLPI + CpG soluble mixtures, a single dose of both evasin/mTSLPI NP and evasin/mTSLPI NP + CpG substantially increased anti-mTSLPI IgG titers at week 3 (Figure S3). Evasin/mTSLPI NP improved the antievasin humoral immune response only when supplemented CpG adjuvants and no soluble formulation induced detectable antievasin titers (Figure S3). This trend held after the boost immunization, where evasin/mTSLPI NP + CpG resulted in the highest IgG, IgG2a, and IgG1 titers against both evasin and mTSLPI on day 49 (Figure 3B,C). Although a single dose of evasin/mTSLPI NP without adjuvant promoted anti-mTSLPI humoral immune response on day 21, a second dose of evasin/mTSLPI NP did not improve either antievasin or anti-mTSLPI IgG titers. These results show that both the nanoparticle presentation of antigens and adjuvant is necessary for inducing robust humoral immune responses against tick salivary proteins. CpG ODN 1826 is an adjuvant that elicits production of proinflammatory cytokines by stimulating TLR-9 in immune cells.^29,30^ However, adjuvants could not enhance humoral immune responses to soluble antigens before or after the boost injection (Figure S3 and Figure 3B,C). We have previously observed that desolvation of antigens into NPs can alter antigen structure using ovalbumin NP.^31^ If evasin structure is changed by desolvation, the immunosuppressive chemotaxis inhibition function could be blunted compared to soluble, nondesolvated antigens or antigens produced by nucleic acid vaccines.

**Figure 3:**
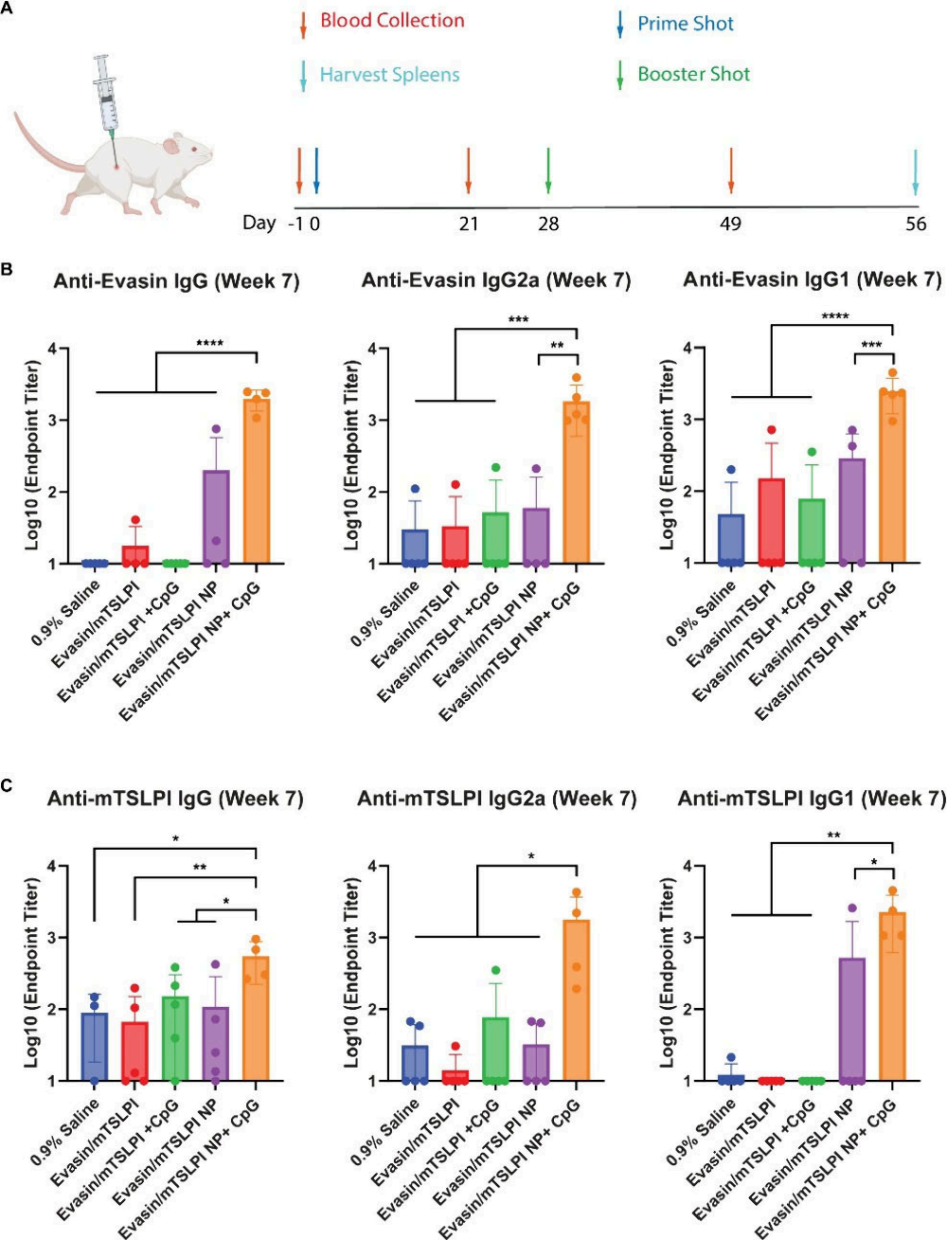
*In vivo* humoral immune responses induced in mice (*n* = 5) by intramuscular vaccination with PBS (control), soluble evasin/mTSLPI mixture, soluble evasin/mTSLPI mixture + CpG, evasin/mTSLPI NP, and evasin/TSLPI NP + CpG. (A) Timeline for *in vivo* study created with BioRender.com. (B, C) Titers of (B) antievasin and (C) anti-mTSLPI IgG, IgG2a, and IgG1 in sera collected at week 7 postprime. p-values were determined by one-way Anova with Turkey’s posthoc multiple comparison analysis: * for ≤ 0.05, ** for ≤ 0.01, *** for ≤ 0.001, **** for *p* ≤ 0.0001.

Th1 and Th2 immunity orchestrates critical crosstalk between immune cells to induce cellular and humoral immune responses against pathogens.^32–34^ While Th2 cells are closely associated with humoral immunity and defense against helminth parasites and extracellular bacteria, Th1 cells trigger cellular immunity and provide host defense against intracellular viruses and bacteria. Given their ability to promote immune responses, their roles in immune responses to pathogens are critical. To evaluate the cell-mediated immune responses elicited by evasin/mTSLPI NP + CpG, we isolated splenocytes from the spleens of immunized mice and restimulated them with soluble evasin or mTSLPI protein for ELISpot analysis. After restimulation, we observed significantly enhanced IFN-γ secreting T cell responses against mTSLPI in mice vaccinated with evasin/mTSLPI NP + CpG while other groups did not improve responses, as shown in Figure 4. This Th1 cellular immune response aligned well with the humoral immune response elicited by evasin/mTSLPI NP + CpG, in particular, the high IgG2a titers. Furthermore, this data is consistent with *ex vivo* activation of primary bone marrow dendritic cells (BMDCs) where evasin/mTSLPI NP + CpG induced the highest expression levels of proinflammatory tumor necrosis factor alpha (TNFα) and IL-12p70 (Figure S4A,B). Interestingly, the secretion of immune regulatory cytokine IL-10 was also upregulated. A similar trend was previously reported via I BMDC stimulation with GM-CSF and TNFα.^35^ The fact that evasin/mTSLPI NP + CpG strongly induced Th1 responses whereas evasin/mTSLPI NP without CpG did not is likely related to the role of CpG ODN 1826 as a Th1 adjuvant that promotes priming of Th1 cells to secrete proinflammatory IFN-γ and IL-12.^29,30,36^ It should be noted that IL-10 functions as a negative feedback mechanism that plays a critical role in maintaining immune homeostasis and limiting excessive inflammatory responses.^37,38^ Therefore, IL-10 secretion induced by evasin/mTSLPI NP + CpG may help mitigating potential side effects, such as tissue damage, that could result from excessive proinflammatory immune responses. Additionally, evasin-responsive IL-4 secreting T cells, which are generally considered to bias toward Th2 responses,^39–41^ were markedly higher in mice immunized with evasin/mTSLPI NP + CpG than other formulations (Figure 4B). mTSLPI-specific IL-4 responses were less clear, with only a statistically significant increase by evasin/mTSLPI NP + CpG over soluble evasin/mTSLPI + CpG. There was a lack of IL-4 secretion from BMDCs (Figure S4C), which were derived from BALB/C, even when they were stimulated by evasin/mTSLPI NP + CpG. DCs themselves are not generally considered to generate IL-4, though IL-4 can be secreted upon their exposure to Th2 antigens or cytokines from other immune cells.^42–44^ Although the mechanism of *in vivo* IL-4 secretion induced by evasin/mTSLPI NP + CpG remains unclear, strong B cell activation can promote polarization of Th2 cells.^33,34,45,46^ Such correlation was also shown in high IgG2a and IgG1 titers induced in mice immunized with evasin/mTSLPI NP + CpG (Figure 3), which are surrogate markers of Th1 and Th2 cellular responses, respectively.^32^ mRNA lipid NPs encoding 19 tick salivary proteins were also previously shown to enhance the expression of both IFN-γ and IL-4 from peripheral blood mononuclear cells (PBMCs) upon restimulation with *I. scapularis* saliva, which was strongly correlated with potent humoral immune responses and prophylactic activities against tick challenge in guinea pigs. This suggests that both potent Th1 and Th2 responses can be elicited by vaccines formulated with multiple tick salivary proteins, which may contribute to tick resistance.^15^

**Figure 4:**
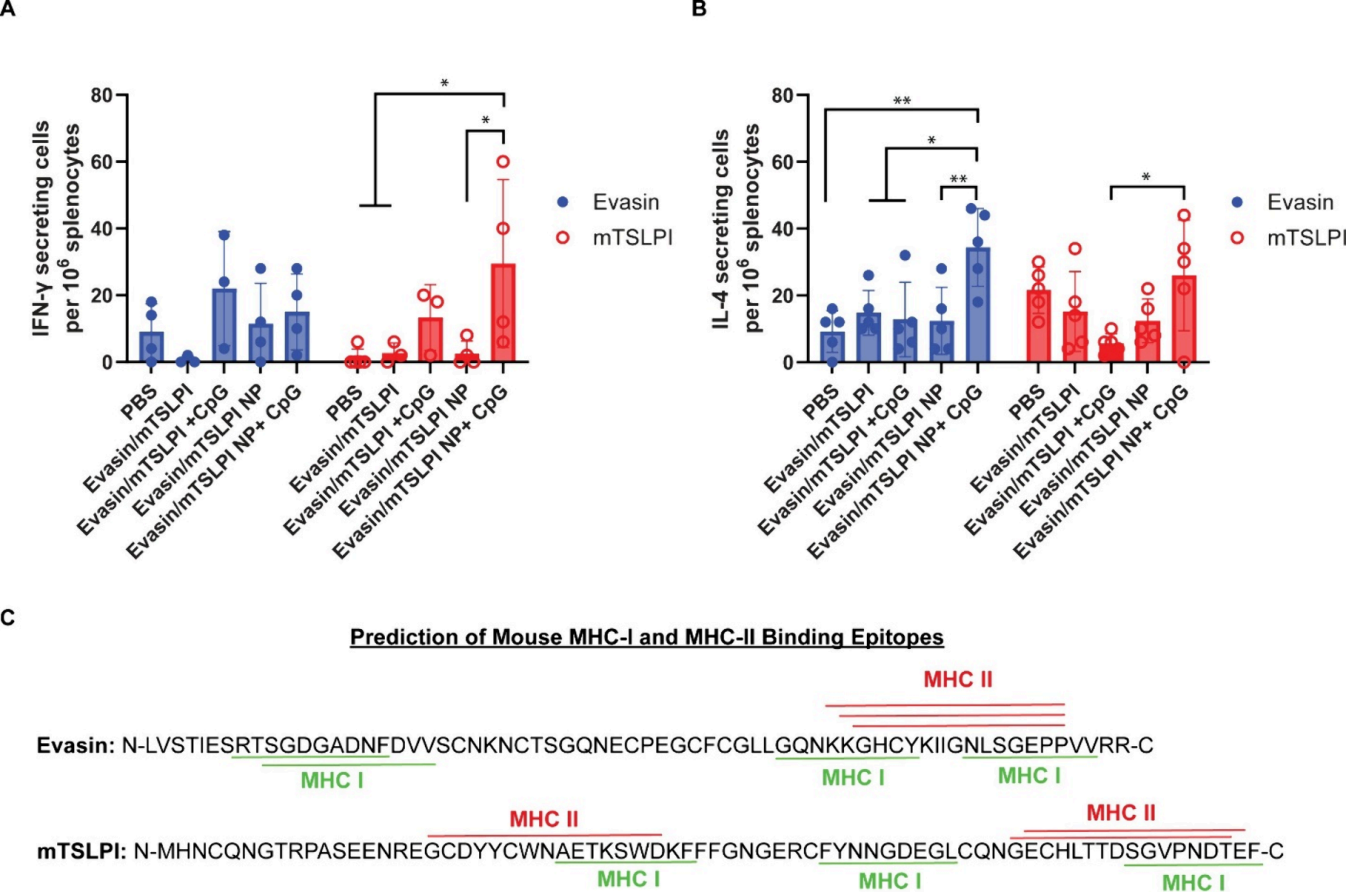
Cellular immune responses induced in mice (*n* = 5) vaccinated with PBS (control), evasin/mTSLPI, evasin/mTSLPI + CpG, evasin/mTSLPI NP, and evasin/TSLPI NP + CpG. (A, B) ELISpot assay was conducted for quantitative analysis of evasin and mTSLPI-responsive (A) IFN-γ and (B) IL-4 secreting splenocytes. P-values were determined by one-way Anova with Turkey’s posthoc multiple comparison analysis: * for ≤ 0.05, ** for ≤ 0.01. (C) Mouse MHC-I and MHC-II binding epitopes were predicted by NetMHCpan 4.1.^47^

In addition to the assessment of cellular immune responses, we analyzed potential T cell epitopes from evasin and mTSLPI that may contribute to cellular immunity. Using etMHCpan4.1,^47^ we predicted potential MHC-I and MHC-II binding epitopes with relatively high prediction scores for both tick salivary proteins. For evasin, the peptides SRT­SG­DG­AD­NF­DVV and KKG­HC­YK­II­GN­LS­GEPP were predicted to bind to MHC-I and MHC-II molecules, respectively, with a high affinity. In the case of mTSLPI, MHC-I and MHC-II binding epitopes were predicted to be clustered near the C-terminus (GEC­HL­TT­DS­GV­PN­DTEF). Interestingly, a MHC-I and MHC-II binding site near N-terminus (GCD­YY­CW­NA­ET­KS­WDKF) shows a high degree of sequence homology with the most immunogenic epitope (GCD­YY­CW­NA­ET­KS­WD­QF­FFG) of Salp14, a tick saliva protein.^48^ Epitopes in evasin tended to exhibit stronger predicted binding to MHC-II (scores >0.90) compared to mTSLPI (<0.20), whereas those in mTSLPI showed higher predicted binding affinity to MHC-I (scores >0.40) than those in evasin (<0.30). These findings suggest differential immunogenic potential between the two tick salivary proteins. Further studies to experimentally identify T cell epitopes from the tick salivary proteins will be a valuable direction for the design of a tick salivary protein-based subunit vaccine in the future.

## Conclusions

In this study, we evaluated the potential of desolvated immunosuppressive tick salivary protein NPs as a vaccine strategy. Tick salivary proteins are novel attractive vaccine candidates, as they can be targeted to inhibit their immunosuppressive activities, promoting the immune system to combat various pathogens introduced from tick bites. This work demonstrated the value of both desolvated NPs and CpG adjuvant to induce humoral and cellular immune responses against tick salivary proteins; soluble tick salivary proteins with or without adjuvant could not elicit measurable immune responses. While other pathogen-derived and model antigens did not require adjuvants when desolvated into NPs,^23,24,31^ the immunosuppressive nature of evasin and TSLPI may be a significant reason for the need for adjuvant to induce enhanced humoral and cellular immune responses. Future work will assess the translational potential of tick saliva NPs by performing challenge studies with disease-carrying ticks.

## Supplementary Material



## Notes

Additional materials and methods, ESI-MS analysis of evasin-3 and mTSLPI after purification, antievasin and anti-mTSLPI IgG week 3 titers, and BMDC cytokine measurement (PDF)
