# Supplementary material for: Evasin and TSLPI Tick Salivary Antigen Subunit Vaccine Nanoparticles Induce Humoral and Cellular Immunity
Source: ACS Nanosci Au. 2025 Aug 12;5(5):337–43. doi: 10.1021/acsnanoscienceau.5c00034 (PMC12531863; doi:10.1021/acsnanoscienceau.5c00034)
Supplement: Supplementary file 1 [file ng5c00034_si_001.pdf]

Evasin and TSLPI Tick Salivary Antigen Subunit Vaccine Nanoparticles Induce Humoral and Cellular Immunity

Jaeyoung Park[a], Thomas Pho[a,b], Stepan S. Denisov[c], Ingrid Dijkgraaf[c,], Julie A. Champion[a,b,*]*

[a]School of Chemical and Biomolecular Engineering, Georgia Institute of Technology, 950 Atlantic Dr. NW, Atlanta, GA, 30332, USA.
[b]Bioengineering Program, Georgia Institute of Technology, Atlanta, GA, 30332, USA.
[c]Department of Biochemistry, Cardiovascular Research Institute Maastricht (CARIM), Maastricht University, Masstricht 6229 ER, Netherlands
[*]co-corresponding authors: julie.champion@chbe.gatech.edu (J.A.C) and i.dijkgraaf@maastrichtuniversity.nl (I.D.)

Materials and Methods

Evasin-3 Production

Evasin-3 Sequence:

LVSTIESRTSGDGADNFDVVSCNKNCTSGQNECPEGCFCGLLGQNKKGHCY KIIGNLSGEPPVVRR

The *C*- and *N*-terminus of Evasin-3 were both synthesized by manual *tert*-butyloxycarbonyl (tBoc) solid-phase peptide synthesis (SPPS) on a 0.25 mmol scale using *in situ* neutralization. O-(1H-6-Chlorobenzotriazole-1-yl)-1,1,3,3-tetramethyluronium hexafluorophosphate (HCTU) as a and *N*,*N*-Diisopropylethylamine (DIPEA) were used for activation of the amino acids. *N*,*N*-dimethylformamide (DMF) was used as a solvent during synthesis and *t*Boc deprotection was performed with trifluoroacetic acid (TFA).

The *N*-terminal fragment (LVSTIESRTSGDGADNFDVVSCNKNCTSGQNECPEG) was synthesized on Boc-leucine-4-hydroxymethyl-phenylacetamidomethyl (PAM) resin. To obtain a thioester, 3-mercaptopropionic acid (MPA) was coupled 2 times for 30 minutes. Deprotection of the trityl-group of MPA was done by treating the resin with a mixture of 95/2.5/2.5 TFA/TIS/H_2O.

The *C*-terminal fragment (CFCGLLGQNKKGHCYKIIGNLSGEPPVVRR) was synthesized on Boc-Arg(Tos)-PAM resin.

After chain assembly, the peptides were deprotected and cleaved from the resin by treatment with anhydrous hydrogen fluoride HF for 1 h at 0 °C, using 4% (v/v) p-cresol as a scavenger. Following cleavage, the peptides were precipitated with ice-cold diethyl ether, dissolved in a H_2O/CH_3CN/TFA mixture and lyophilized.

Native chemical ligation of unprotected synthetic peptide segments was performed as follows: 0.1 M TRIS buffer, pH 8, containing 6 M Gnd-HCl was added to dry peptides yielding approx. 10 mg/mL of peptide fragments. Subsequently, 1% (v/v) benzyl mercaptan and thiophenol were added. The ligation reaction was performed in a heating block at 37 ºC and the mixture was vortexed periodically to equilibrate the thiol additives. Reaction progress was analyzed on analytical HPLC and ESI/MS or with UPLC-MS. Ligated material was purified by HPLC, lyophilized and subjected to oxidative folding. Oxidative folding of the proteins was performed in

0.1 M TRIS buffer, pH 8.0, containing 1 M Gnd-HCl, and 1 mM cystine/8 mM cysteine as a redox couple. The reaction mixture was stirred at 4 °C, and after reaction completion, as detected with analytical HPLC, purified using a (semi)preparative HPLC system comprised of a Waters Deltaprep System consisting of a Waters Prep LC Controller and a Waters 2487 Dual wavelength Absorbance Detector (λ = 214 nm).

mTSLPI Expression and Purification

mTSLPI Sequence:

MHNCQNGTRPASEENREGCDYYCWNAETKSWDKFFFGNGERCFYNNGD
EGLCQNGECHLTTDSGVPNDTEF

The pET23a vector containing the complementary DNA of the TSLPI protein was purchased from GenScript, Table S1. The protein was expressed in BL21 (DE3) Star (Novagen). The cells were grown in 1 liter of standard LB medium at 37 °C in the presence of the required antibiotic. Once the A600 value reached 0.6–0.8, expression of protein was induced with 0.1 isopropyl β-D-1-thiogalactopyranoside (Sigma–Aldrich). The cells were harvested 3 h after induction by centrifugation at 4,000 rpm for 20 min at 4 °C. The bacterial pellet was resuspended in 6 M Gdn–HCl, 50 mM Tris, and pH 8 at a concentration of 1 mg/ml and stirred for 1 h at RT. Then cell debris was removed by centrifugation at 10,000 rpm for 20 min at 4 ℃, and the soluble fraction was dialyzed overnight against 0.5% acetic acid using a 3.5 kDa Spectra/Por RC membrane (Repligen). After dialysis, the soluble fraction was separated by centrifugation at 10,000 rpm for 20 min at 4 ℃ and lyophilized.

Lyophilized material was dissolved in 6 M Gdn–HCl, 0.1 mM Tris, pH 8, at 20 mg/ml and then added dropwise to 1 M 6 M Gdn–HCl, 0.1 mM Tris, pH 8, 10 mM cysteine, 1 mM cystine, at 4 °C to a final concentration of 1 mg/ml. The folded protein was purified by HPLC using 22-mm 250-mm Vydac C18 columns, analyzed by LC-MS, and lyophilized.

Table S1. ORF sequences used for protein expression

Vector name	ORF
TSLPI<br>pET30a<br>*NdeI-HindIII*	cat atg cat aac tgc cag aac ggc acc cgt ccg gcg agc gaa gag aag cgt gaa ggc tgc<br>H M H N C Q N G T R P A S E E K R E G C<br>gac tac tac tgc tgg aat gcg gag acc aat agc tgg gac aag ttc ttt ttc ggt aac ggc<br>D Y Y C W N A E T N S W D K F F F G N G<br>gag cgt tgc ttt tac aac gat ggt ggc gaa ggt ctg tgc caa aac ggc gaa tgc cac ctg<br>E R C F Y N D G G E G L C Q N G E C H L<br>acc acc gat agc agc gtt ccg aat gat agc gat gtg taa aag ctt<br>T T D S S V P N D S D V - K L

Endotoxin was removed from the samples using Pierce High Capacity Endotoxin Removal Spin Columns (Thermo Fisher Scientific) according to manufacturer's instructions.

Nanoparticle Synthesis

Evasin/mTSLPI NPs, evasin NPs, and mTSLPI NPs were synthesized by desolvating 1:1 mixture of evasin and mTSLPI by mass, evasin, and mTSLPI, respectively. Briefly, 400 µL ethanol

was added dropwise to 100 μL of 1 mg/mL protein in phosphate buffered saline (PBS) at a rate of 1 mL/min with a syringe pump while stirring at a speed of 600 rpm. The desolvated NPs were then stabilized by addition of 2.5 μL of 10 mg/mL 3,3'-dithiobis(sulfosuccinimidyl propionate) (DTSSP) crosslinker (Thermo Fisher Scientific) for 1 hr at 25℃. After desolvation and crosslinking, the desolvated NPs were centrifuged at 16,000 xg for 25 min at 4℃ and resuspended in 300 μL of PBS. The NPs were resuspended by pipetting and sonication for 1 sec on and 3 sec off at 50% amplitude 15 times on ice.

Nanoparticle Characterization

The size, polydispersity index (PDI), and zeta potential of the desolvated NPs were evaluated by dynamic light scattering (DLS) with a Malvern Zetasizer Nano ZS90 (Malvern Panalytical). Three DLS measurements of 15 runs were performed per each sample at a scattering angle of 173° with a beam wavelength of 633 nm. In addition, for DLS analysis of NPs, protein was selected as a material with a refractive index of 1.45. PBS was used as a medium for DLS analysis with a viscosity of 0.8882 cP. Prior to zeta potential measurement, desolvated NPs were resuspended in 0.1x PBS, and the Smoluchowski approximation was applied to measure the surface charges of NPs. Transmission electron microscopy (TEM) was used to image the desolvated NPs. 5 μL of the desolvated NPs was loaded onto a 300-mesh carbon film supported copper grid (MilliporeSigma) and incubated for 10 min at 25℃. After washing with deionized (DI) water, the carbon grid was stained by dipping in 5 μL of 1% phosphotungstic acid (MilliporeSigma) solution for 15-20 s, followed by wicking off excess liquid with a Kimwipe and washed with DI water. The TEM samples were then allowed to dry overnight at 25℃. TEM images were taken at 100 kV using a JEOL 100 CX-II TEM.

Animal Vaccination and Organ Harvesting

Five BALB/c mice (6- to 8-week-old, female, Jackson Laboratory) were intramuscularly immunized in the thigh muscles of the hind limb with 50 μL of saline, 10 μg of soluble evasin/mTSLPI mixture (5 μg of each antigen) with and without 10 μg of CpG ODN 1826 in 50 μL saline, or 10 μg of evasin/mTSLPI NPs (5 μg of each antigen) with and without 10 μg of CpG ODN 1826 in 50 μL saline at day 0 and 4 weeks later. Serum antibodies were obtained 3 weeks after each immunization via submandibular blood collection from mice that were anesthetized with 3-5% isoflurane. At the end of the study (8th week), mice were euthanized by CO_2 asphyxiation to collect spleens for ELISpot. Spleens were triturated and filtered through 70 μm strainers with a 1 mL syringe plunger to obtain single cells. After rinsing with complete RPMI 1640 medium supplemented with 10% fetal bovine serum (FBS, Gibco), the single cells were centrifuged at 350 xg, 4℃ for 5 min, resuspended in RPMI 1640 medium with 90% FBS, and were banked at -80℃ prior to ELISpot assay. All animal studies were implemented in accordance with regulations and guidelines approved by Institutional Animal Care and Use Committee at the Georgia Institute of Technology under approved protocol number A100467.

ELISA for Antibody Titer Measurement

ELISA was performed to determine evasin- or mTSLPI-specific antibody titers in sera from immunized mice. Nunc Maxisorp 96-well immune assay plates (Thermo Fisher Scientific) were coated with 100 μL/well of 1 μg/mL evasin or mTSLPI in PBS overnight at 25℃. Each well was washed three times with 200 μL/well of washing buffer (PBS, pH 7.4, 0.05 % Tween-20) (Thermo Fisher Scientific). 200 μL/well of blocking solution (PBS, pH 7.4, 1% BSA) (Thermo

Fisher Scientific) was added at 25°C for 2 hrs. After washing three times, plates were incubated with serially diluted sera at 25°C for 1 hr before washing. 100 µL/well of 1:5,000 diluted HRP-conjugated goat anti-mouse IgG (Southern Biotech), IgG1 (Southern Biotech), or IgG2a (Southern Biotech) was added to each well for 1 hr, followed by washing three times. 50 µL/well of 1-Step™ TMB ELISA Substrate Solution was added to develop color. The enzymatic activity of HRP was quenched by 50 µL/well of ELISA Stop Solution, and the absorbance at 450 nm and 570 nm was measured using BioTek Synergy HTX Multimode Reader (Agilent).

ELISpot for Cellular Immune Response

Cellular immune responses were analyzed by using Mouse IFN-γ/IL-4 Double-Color ELISPOT (ImmunoSpot) as per manufacturer's instructions. Briefly, 15 µL of 70% ethanol was added into each well of a 96-well, high-protein-binding, PVDF filter plate, followed by washing with 150 µL PBS. The plate was then coated with 80 µL/well of Mouse IFN- γ/IL-4 Capture Solution. On the next day, Capture Solution was removed from the plate, and the plate was washed with 150 µL PBS. Isolated splenocytes were counted using Vi-CELL BLU Cell Viability Analyzer (Beckman Coulter) and seeded at 2x10^5 cells/well (2x10^6 cells/mL) in the plate and stimulated with evasin or mTSLPI at a final concentration of 10 µg/mL in CTL-Test Medium supplemented with 2 mM L-glutamine (Gibco). After incubation at 37°C for 24 hours, the plate washed with 200 µL of washing buffer three times. The plate was then incubated with 80 µL/well Anti-mouse IFN-γ or IL-4 Detection Solution at 25°C for 2 hrs and washed with 200 µL of washing buffer three times. Afterward, 80 µL/well of Tertiary Solution was added, followed by incubation at 37°C for 1 hr. The plate was washed twice with 200 µL of washing buffer and sequentially twice with 200 µL of DI water. The colors of IFN-γ (blue spot) and IL-4 (red spot) were then developed by Blue Developer Solution and Red Developed Solution. Finally, the plate was rinsed with tap water and allowed to air-dry for 24 hours. IFN-γ and IL-4 spots developed on the membrane of the plate were imaged and counted by BioTek Cytation 7 Cell Imaging Multimode Reader (Agilent).

Isolation and Culturing of Primary Bone Marrow Dendritic Cells

Bone marrow cells were collected for *ex vivo* study by flushing the femurs and tibiae of naïve BALB/C mice with complete RPMI 1640 medium supplemented with 10% fetal bovine serum (FBS, Gibco). The cells were then strained through 70 µm strainers to obtain single cells and resuspended in complete RPMI 1640 medium at 5x10^6 cells/mL. Cells were supplemented with 10% FBS and Granulocyte-macrophage colony-stimulating factor (GM-CSF, Invitrogen) at a final concentration of 20 ng/mL and continually cultured for 5 days. Light microscopy was used to confirm the differentiation into bone marrow dendritic cells (BMDCs) by observing the dendritic features of cells.

ELISA for Cytokine Measurement

BMDCs are loosely adherent and were collected by centrifugation at 400 x g for 10 mins. Cells were counted using Vi-CELL BLU (Beckman Coulter) and plated at 5x10^5 cells/well (2.5 x10^6 cells/mL) in a 24-well, round bottom plate. BMDCs were stimulated with 10 µL of PBS, 2.5 µg/well of soluble evasin/mTSLPI with and without 2.5 µg/well of CpG ODN 1826, or evasin/mTSLPI NPs with and without CpG ODN 1826 in 10 µL of PBS for 24 hours. The cytokines released from BMDC were collected from supernatant after centrifugation at 400 xg for 10 min. Secreted cytokines were measured for IL-4, TNFα, IL-10, and IL-12 using DuoSet® ELISA kits (R&D Systems) as per manufacturer's protocol. After color development by TMB

ELISA Substrate Solution (Thermo Fisher Scientific) and ELISA Stop Solution (Invitrogen), the absorbance was measured at 450 nm and 570 nm using BioTek Synergy HTX Multimode Reader (Agilent).

Statistical Analysis

One-way ANOVA comparison with Turkey's post-hoc multiple comparison analysis was used for statistical analysis. Statistical significance was denoted as follows: ns for statistically non-significant differences, * for $p \leq 0.05$, ** for $p \leq 0.01$, *** for $p \leq 0.001$, **** for $p \leq 0.0001$. All plotted data are presented with error bars, indicating mean values with standard deviation. The statistical analysis was carried out using Graphpad Prism 10. Additionally, outliers were identified and excluded from ELISpot data based on a multiple outlier detection ROUT analysis (Q = 5%) of raw data[1] via Graphpad Prism 10.

Supplementary Figures

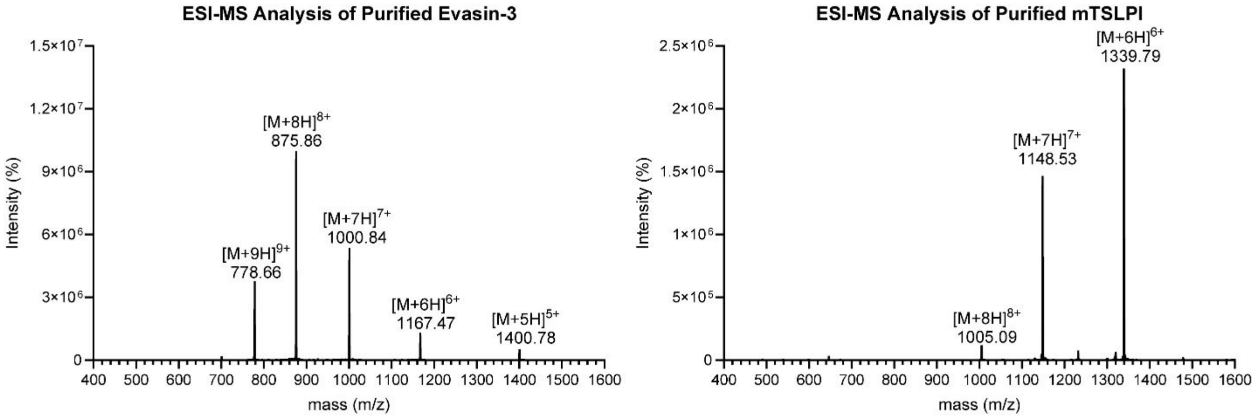

Figure S1. ESI-MS analysis of evasin-3 and mTSLPI after purification. ESI-MS spectra were deconvoluted to determine molecular weights of evasin-3 (6,995.80 Da) and mTSLPI (8,028.68 Da).

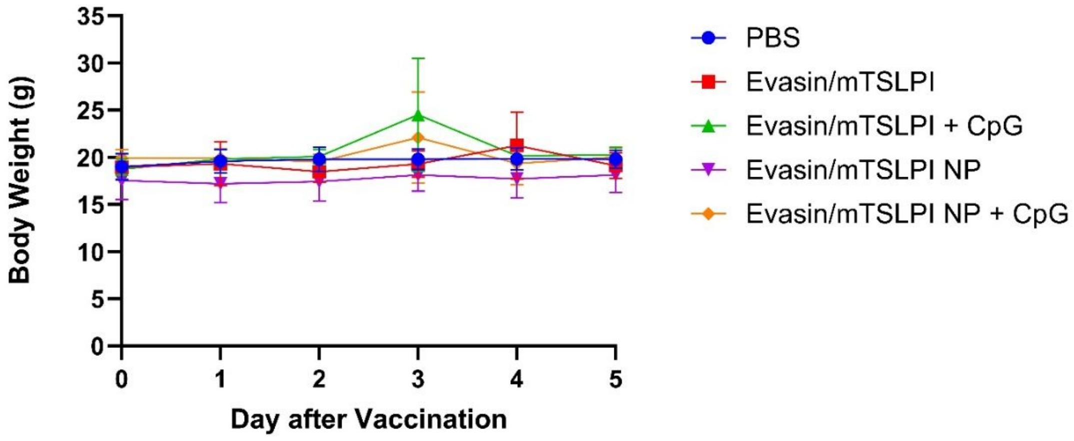

Figure S2. Body weight change of mouse (n=5) after vaccination with PBS (control), evasin/mTSLPI, evasin/mTSLPI + CpG, evasin/mTSLPI NP, and evasin/mTSLPI NP + CpG.

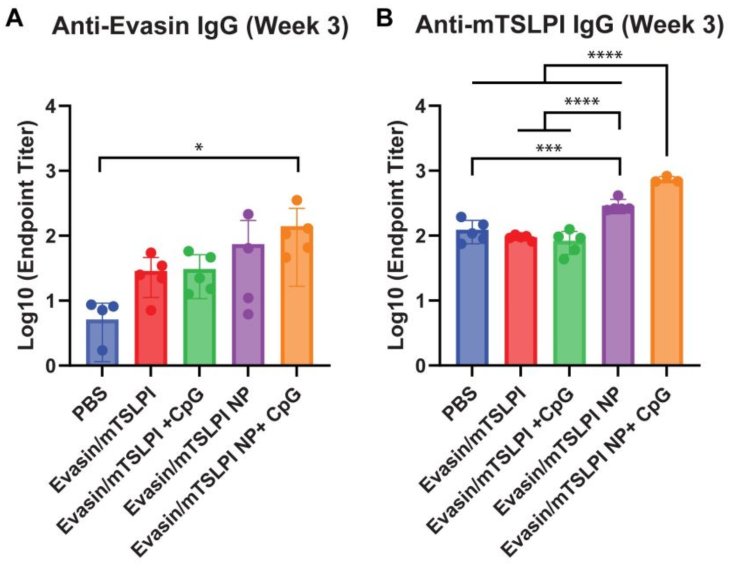

Figure S3. Anti-evasin and anti- mTSLPI IgG titers in mouse sera (n=5) collected at 3 weeks after prime vaccination with PBS (control), evasin/mTSLPI, evasin/mTSLPI + CpG, evasin/mTSLPI NP, and evasin/mTSLPI NP + CpG.

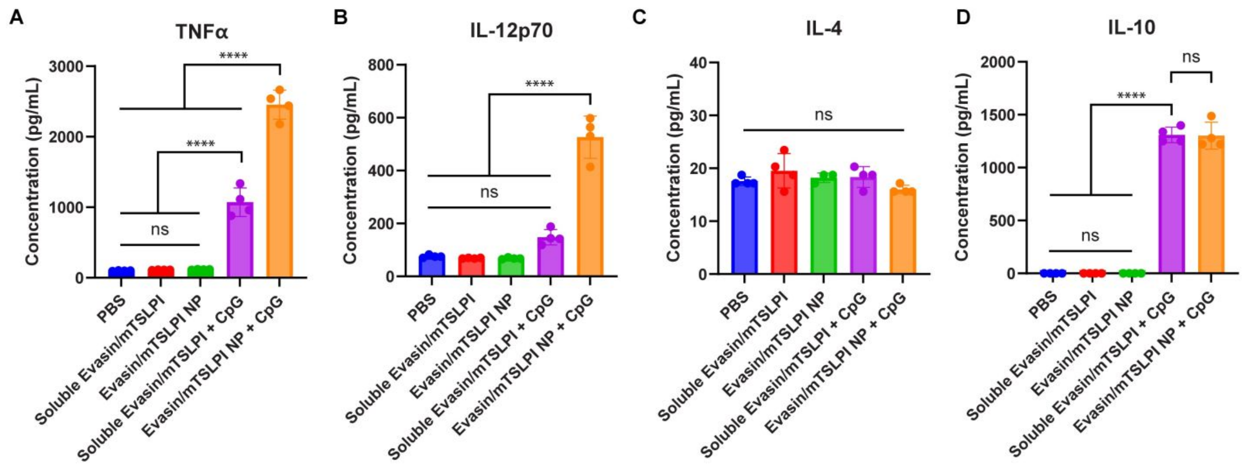

Figure S4. ELISA was performed to assess cytokine levels of (A) TNFα, (B) IL-12p70, (C) IL-4, and (D) IL-10 secreted from BMDCs stimulated with PBS (control), evasin/mTSLPI, evasin/mTSLPI NP, evasin/mTSLPI + CpG, and evasin/mTSLPI NP + CpG.

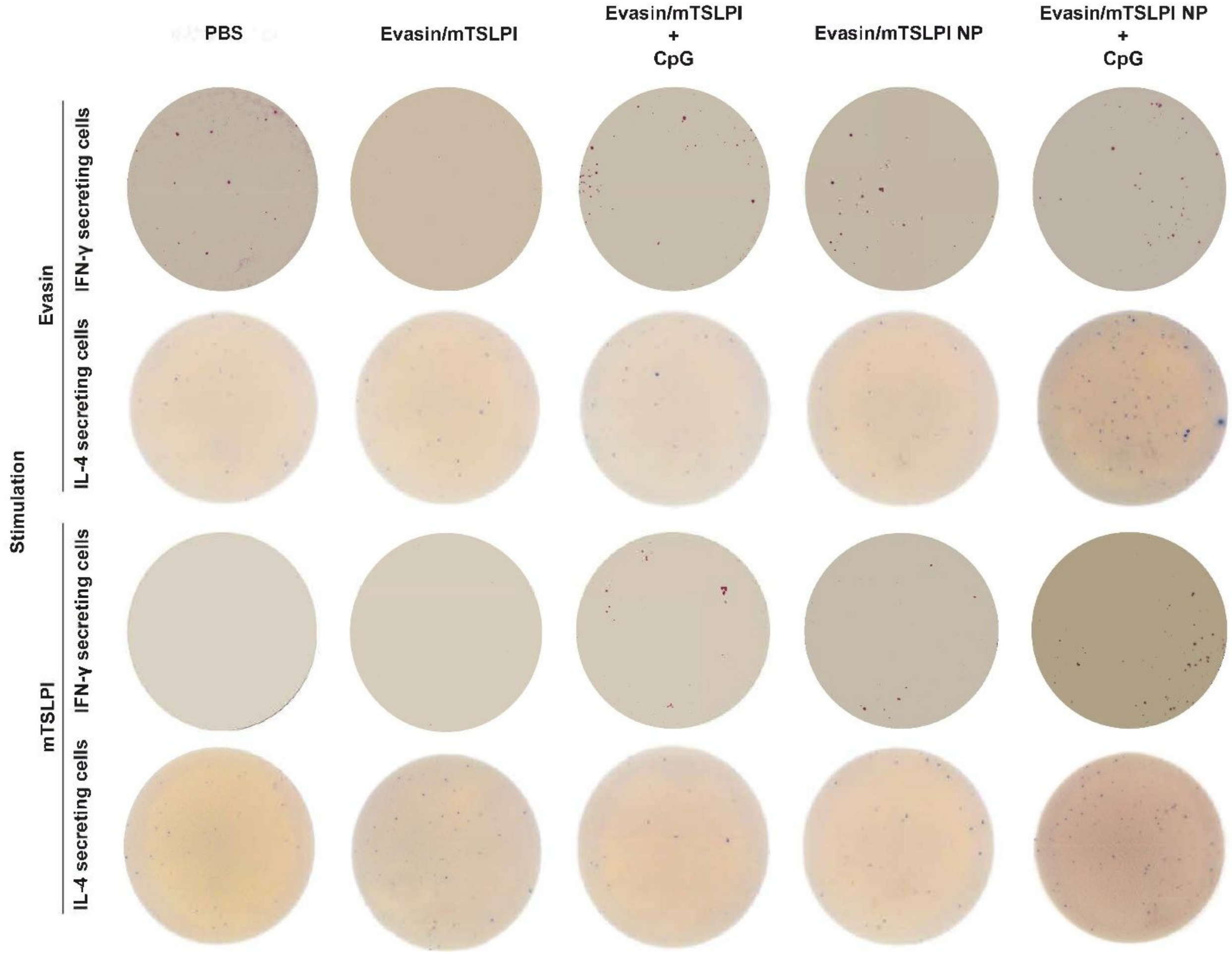

Figure S5. Representative ELISpot images of evasin and mTSLPI-responsive IFN-γ (red spot) and IL-4 (blue spot) secreting splenocytes harvested from vaccinated mice (n=5).

References

(1) Motulsky, H. J.; Brown, R. E. Detecting outliers when fitting data with nonlinear regression – a new method based on robust nonlinear regression and the false discovery rate. *BMC Bioinformatics* **2006**, *7* (1), 123. DOI: 10.1186/1471-2105-7-123.
